# UAV-Assisted Covert Communication with Dual-Mode Stochastic Jamming

**DOI:** 10.3390/s26020624

**Published:** 2026-01-16

**Authors:** Mingyang Gu, Yinjie Su, Zhangfeng Ma, Zhuxian Lian, Yajun Wang

**Affiliations:** 1 Ocean College, Jiangsu University of Science and Technology, Zhenjiang 212000, China; 17625312888@163.com (M.G.); zhuxianlian@just.edu.cn (Z.L.); wangyj1859@just.edu.cn (Y.W.); 2 College of Electronic and Information Engineering, Nanjing University of Aeronautics and Astronautics, Nanjing 211106, China; zhangfeng.ma@vip.126.com

**Keywords:** covert communication, UAV, dynamic trajectory planning, average covert rate

## Abstract

Covert communication assisted by unmanned aerial vehicles (UAVs) can achieve a low detection probability in complex environments through auxiliary strategies, including dynamic trajectory planning and power management, etc. This paper proposes a dual-UAV scheme, where one UAV transmits covert information while the other one generates stochastic jamming to disrupt the eavesdropper and reduce the probability of detection. We propose a dual-mode jamming scheme which can efficiently enhance the average covert rate (ACR). A joint optimization of the dual UAVs’ flight speeds, accelerations, transmit power, and trajectories is conducted to achieve the maximum ACR. Given the high complexity and non-convexity, we develop a dedicated algorithm to solve it. To be specific, the optimization is decomposed into three sub-problems, and we transform them into tractable convex forms using successive convex approximation (SCA). Numerical results verify the efficacy of dual-mode jamming in boosting ACR and confirm the effectiveness of this algorithm in enhancing CC performance.

## 1. Introduction

In addition to technologies like extremely large-scale array and intelligent reflecting surface (IRS) [1,2], covert communication (CC) serves as a core complement in 6G, essential for its integrated security architecture. CC offers a novel secure transmission pathway, distinct from traditional cryptography, physical-layer security technologies, and authentication methods. Building on the foundational research of the square root law introduced in [3], extensive research has been conducted on CC recently. The authors in [4] defined a new metric to evaluate the covertness, which was utilized to examine an achievable rate for CC with noise uncertainty. In [5,6,7], relay selection was considered in CC and the impact of system parameters on performance was analyzed. As stated in [8], the authors first proposed a new CC scheme with a distance-dependent relay selection strategy. Different from the existing relay selection strategies, this scheme jointly considers the locations of the source and the destination as the criteria for relay selection. In [9], the authors investigated CC with the assistance of relay in cognitive radio networks. They proposed a novel cooperative jamming scheme, where the relay and source, respectively, undertook the functions of signal transmission or jamming at different transmission stages, and the impacts of parameters on the effective covertness rate, outage probability, and detection error probability (DEP) were analyzed. In [10], the authors studied a multi-antenna half-duplex AF relay-assisted covert communication system, focusing on two scenarios with instantaneous eavesdropping channel state information (CSI) and statistical eavesdropping CSI. The research found that this system can achieve significantly performance gains in both scenarios. In [11], the authors proposed a joint optimization strategy for power and time slot allocation in a covert communication system with half-duplex relaying. It was shown that the strategy can improve the effective number of transmission bits. In [12], the authors constructed a covert communication system which does not require collaboration with a jammer. By combining semantic information and power control and designing efficient reinforcement learning algorithms, the covertness and quality of information transmission was improved. CC with the uncertainties of location and noise was investigated in [13,14], respectively. A covert communication system assisted by a cognitive jammer was investigated in [15]. It proved that compared to a traditional non-informed jammer, the cognitive jammer can achieve higher covert rates. On this basis, the authors in [16] conducted further research on the cognitive jammer, by analyzing the impact of various jamming strategies on the system performance. IRSs use an array of reflective elements to steer electromagnetic waves toward a desired direction, which can enhance the system performance [17]. Recently, it has attracted a great deal of attention from researchers in the field of CC. In [18], the authors investigated CC for an IRS-assisted system and showed that IRSs can efficiently improve system performance. The authors in [19] proposed a novel CC scheme with finite block length. The authors designed two working modes using an IRS and intelligent omni-surface. The scheme can dynamically switch between the two modes to achieve covert transmission in the Non-Orthogonal Multiple Access (NOMA) network and improve CC performance. In [20], the authors investigated IRS-assisted covert communication with a limited alphabet input. The impacts of transmission outage probability, DEP, and the reflection coefficient on system performance were analyzed. Similar to IRSs, backscatter communication is also a key technology that can reconstruct the wireless communication environment in the field of CC. However, in the existing research studies, the inevitable residual hardware impairments (RHIs) in RF transceivers have mostly been ignored. In [21], the authors investigated backscattering CC in the presence of RHI. In this case, the communication performance was evaluated through the analysis of the channel. Moreover, the authors optimized the reflection coefficient of power and detection to enhance the covert performance. In [22], the authors studied the covert backscatter communication system with hardware impairments. Unlike the continuous artificial noise emission, the authors proposed an intermittent artificial noise-assisted strategy, which can obtain better covertness and reliability. In [23], the authors first applied the Rate Split Multiple Access (RSMA) technique to the environmental backscatter communication system. RSMA can achieve better covert performance compared to the NOMA scheme. The multi-channel can have a positive impact on covertness. In [24], the authors combined the uncertainty of random sub-channel selection and artificial noise to confuse the detection of the eavesdropper. In [25], the authors established a multi-channel model to enhance the covert performance, and it shows that the average effective throughput is maximized through optimization of sub-channel allocation and power. The rise in integrated sensing and communication (ISAC) technology provides methodological support for the realization of covert communication. In [26], the authors used a dual-function radar to simultaneously perceive the eavesdropper and conduct covert communication with a legitimate target. By solving a beamforming optimization problem, it efficiently counters the detection of the eavesdropper and improves the covert throughput. To address the threat of eavesdroppers with adaptive detection thresholds, the authors in [27] proposed a CC framework based on ISAC. This framework enabled a dual-function system to communicate with ground users and simultaneously sense aerial targets. The sensing beamformers were optimized to achieve the maximum covert rate. In [28], the authors designed a dual-function source which was able to sense the target of the eavesdropper and then transmit jamming signals with various powers to confuse its detection. The performance was analyzed considering both the imperfect CSI and statistical CSI scenarios. The authors in [29] explored a dual-mode scheme utilizing ISAC. The communication process comprised two stages. The full-duplex transceiver acted as the receiver and performed sensing and jamming, respectively, in the two stages to improve the covert rate. In [30], the authors presented a robust beamforming scheme for the CC system with ISAC to address the challenges brought on by the imperfect CSI.

Recently, UAVs are increasingly utilized across various applications due to their high maneuverability, low cost, and advantages associated with line-of-sight (LoS) links [31,32,33,34]. UAVs act as mobile base stations, relaying source signals to designated destinations. Furthermore, UAV communication networks can provide greater coverage and communication capacity compared to traditional ground wireless communication networks. In [35], the authors introduced a finite block-length transmission model for the first time in an IRS-aided multi-antenna UAV CC system. An optimization method considering trajectory, beamforming, and intelligent reflector phase shift was proposed; then, an algorithm was developed for reducing the complexity of the problem. In [36], the authors proposed a long-distance covert communication scheme utilizing UAV relays with a constrained block length. The authors in [37] proposed a multi-hop relay strategy by designing a coding rate and multi-hop routing to guard against UAVs’ surveillance. The authors in [38] utilized a UAV as a jammer and proposed a geometric method for optimizing the UAV’s position, which is able to achieve better performance with lower complexity. In [39], the authors developed a collaborative anti-eavesdropping strategy that can maximize covert performance by transmitting covert information to a specified location using two UAVs. The authors in [40] investigated a multi-UAV covert communication system and proposed a joint dynamic scheduling and sensing jamming scheme to achieve better covert performance. The authors in [41] considered the impact of ground obstacles on air-to-ground channels and proposed an obstacle-aided UAV CC scheme. In [42], the authors studied CC between UAVs and balanced the covertness and reliability of the system. In [43], the authors proposed a robust CC paradigm with a multi-antenna UAV jammer. In this framework, the transmitter employed maximum ratio transmission, while the UAV jammer utilized zero-forcing beamforming to confound the warden without impairing the legitimate link. It is shown that the scheme is able to improve the covert rate while complying with strict undetectability and anti-eavesdropping requirements. To enhance the anti-jamming capability of UAVs, the authors in [44] proposed a system with dual transmitting antennas and dual receiving antennas. This system can utilize the phase difference in the signals transmitted by the dual antennas to construct a covert channel. The authors in [45] investigated the timeliness of short packet covert communication assisted by UAV relay. A two-stage hybrid air–ground cooperative detection scheme was proposed in [46]. The authors jointly optimized the UAV’s position and beamforming pattern to enhance the communication performance. In [47], the authors studied a covert and secure communication mechanism with untrusted UAV relay. By meticulously designing jamming and power control strategies, the eavesdropping by untrusted relay was prevented, thus achieving secure and reliable communication in a complex wireless environment. In [48], the authors addressed the challenge of achieving reliable and covert communication with both passive and active wardens and proposed a novel UAV-relaying CC framework. In [49], the authors studied air-to-ground CC. With the assistance of ground jammers, the covert rate was maximized through the optimization of trajectory and power. In [50], with the condition that the DEP is constrained, the minimum average covert rate (ACR) was enhanced through adjusting the flight trajectory of the UAV. In [51], the authors studied the trajectory optimization problem for UAV-aided CC with time-varying channels, aiming to maximize the total system throughput under covert constraints. In [52], a covert communication system supported by two UAVs was proposed, where one UAV facilitates communication with ground users, and the other functions as a friendly jammer to disrupt the detection by the eavesdropper. The covert performance was optimized by optimizing the flight speeds, powers, and time slots.

This paper focus on a CC system configured with dual UAVs. One (i.e., UAV-A) is responsible for transmitting covert information to legitimate receivers, while the other (i.e., UAV-J) disrupts the detection of the eavesdropper through artificial noise (i.e., jamming) with uniformly distributed power. Different from the traditional single-mode stochastic jamming [38,40,49,52], we propose a dual-mode jamming scheme, i.e., whether UAV-A transmits covert information or not, UAV-J will generate uniformly distributed artificial noise at varying power levels. Joint optimization is constructed to maximize the ACR, by optimizing the flight speeds, accelerations, power, and trajectories of the dual UAVs. This is a non-convex problem; hence, we develop an algorithm to solve it. Specifically, we decompose the optimization into three sub-problems and transform the sub-problems into tractable convex forms using a successive convex approximation (SCA) technique. Numerical results indicate that dual-mode stochastic jamming is able to significantly improve the ACR, and the algorithm can efficiently enhance the performance of CC.

The following sections describe the remainder of this paper. In Section 2, the system model is described. We propose a novel dual-mode jamming scheme and then analyze the detection performance. In Section 3, the problem of maximizing the ACR is formulated, for which we develop an efficient algorithm with SCA. Section 4 presents the numerical results, and Section 5 concludes the paper.

## 2. System Model

### 2.1. System Model

**Figure 1:**
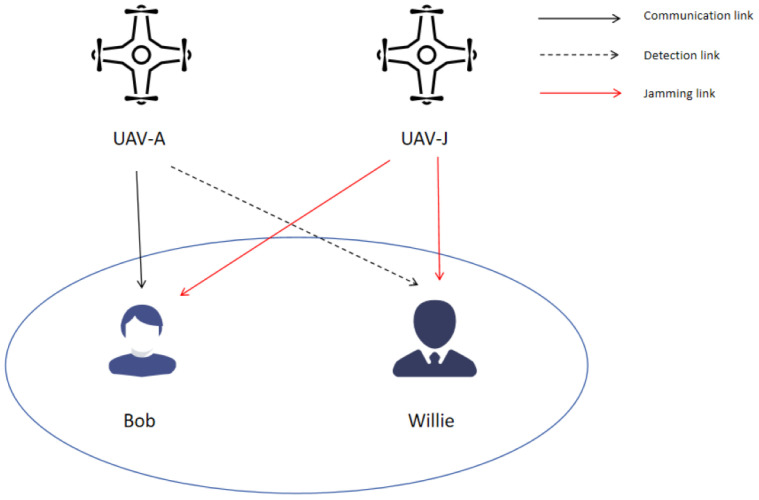
CC system model with dual UAVs.

Figure 1 shows the considered CC system. It can be observed that UAV-A serves as a transmitter, transmitting covert information to a legitimate recipient, i.e., Bob, located on ground. Concurrently, an eavesdropper, i.e., Willie, attempts to detect CC between UAV-A and Bob. In this scenario, UAV-J functions as the mobile jamming unit, continuously emitting jamming signals to disrupt Willie’s detection and facilitate covert communication.

The ground coordinates of Bob and Willie can be represented as $b=[x_{b},y_{b}]$ and $w=[x_{w},y_{w}]$, respectively. The entire flight cycle T is partitioned into N time slots. Each time slot is $\delta_{t}=T/N$, which is assumed to be sufficiently short. For the n-th time slot ($n\in \left\{1,2,\ldots ,N\right\}$), the horizontal positions of the two UAVs are denoted by $a\left[n\right]=[x_{a}\left[n\right],y_{a}\left[n\right]]$ and $j\left[n\right]=[x_{j}\left[n\right],y_{j}\left[n\right]]$, respectively. The movement of the UAVs between two adjacent time slots can be considered as a linear motion. The formula for uniform acceleration linear motion displacement is introduced. In the n-th time slot, let $V_{a}\left[n\right]=\left[V_{xa}\left[n\right],V_{ya}\left[n\right]\right]$ be the speed of UAV-A, $V_{j}\left[n\right]=\left[V_{xj}\left[n\right],V_{yj}\left[n\right]\right]$ be the speed of UAV-J, $A_{a}\left[n\right]=\left[A_{xa}\left[n\right],A_{ya}\left[n\right]\right]$ be the acceleration of UAV-A, and $A_{j}\left[n\right]=\left[A_{xj}\left[n\right],A_{yj}\left[n\right]\right]$ be the acceleration of UAV-J. Therefore, we can have

(1) $$\begin{array}{cc} a[n] & =a\left[n-1\right]+V_{a}\left[n\right]\delta_{t}+\frac{1}{2}A_{a}\left[n\right]\delta_{t}^{2},\;\;n=\left\{2,\ldots ,N\right\}, \end{array}$$

(2) $$\begin{array}{cc} j[n] & =j\left[n-1\right]+V_{j}\left[n\right]\delta_{t}+\frac{1}{2}A_{j}\left[n\right]\delta_{t}^{2},\;\;n=\left\{2,\ldots ,N\right\}, \end{array}$$

(3) $$\begin{array}{c} V_{a}\left[n\right]=V_{a}\left[n-1\right]+A_{a}\left[n\right]\delta_{t},\;\;n=\left\{2,\ldots ,N\right\}, \end{array}$$

(4) $$\begin{array}{c} V_{j}\left[n\right]=V_{j}\left[n-1\right]+A_{j}\left[n\right]\delta_{t},\;\;n=\left\{2,\ldots ,N\right\}. \end{array}$$

The start and end positions of UAVs are expressed as

(5) $$\begin{array}{c} a\left[1\right]=a_{I},a\left[N\right]=a_{F}, \end{array}$$

(6) $$\begin{array}{c} j\left[1\right]=j_{I},j\left[N\right]=j_{F}. \end{array}$$

For the n-th time slot, denoting the transmit power of UAV-A as $P_{a}\left[n\right]$, it should satisfy

(7) $$\begin{array}{c} 0\leq P_{a}\left[n\right]\leq P_{a}^{max}, \end{array}$$

where $P_{a}^{max}$ represents maximum transmit power of UAV-A.

### 2.2. Dual-Mode Jamming Scheme

We assume that the jamming power is random and uniformly distributed. Let $H_{0}$ represent the null hypothesis, indicating that UAV-A does not transmit. Conversely, $H_{1}$ represents that UAV-A does send covert information. Different from the traditional jamming scheme, we propose a dual-mode jamming scheme, where the jamming power emitted by UAV-J follows distinct uniform distributions according to the transmission state of UAV-A (assuming that UAV-A can share its transmission state with UAV-J), i.e.,

(8) $$\begin{array}{cc} H_{0}:f_{P_{j0}\left[n\right]}\left(x\right) & =\left\{\begin{array}{cc} \frac{1}{P_{a}\left[n\right]+P_{j}^{\max}}, & 0\leq x\leq P_{a}\left[n\right]+P_{j}^{\max}\\ 0, & \mathrm{otherwise}, \end{array}\right. \end{array}$$

(9) $$\begin{array}{cc} H_{1}:f_{P_{j1}\left[n\right]}\left(x\right) & =\left\{\begin{array}{cc} \frac{1}{P_{j}^{\max}}, & 0\leq x\leq P_{j}^{\max}\\ 0, & \mathrm{otherwise}, \end{array}\right.\; \end{array}$$

where $P_{j}^{max}$ represents the maximum transmit power of UAV-J for $H_{1}$.

To be specific, when UAV-A does not transmit, i.e., $H_{0}$, the jamming power $P_{j0}$ is uniformly distributed over the interval $\left[0,P_{a}\left[n\right]+P_{j}^{max}\right]$, as seen in (8). When UAV-A transmits covert information, i.e., $H_{1}$, the jamming power $P_{j1}$ follows a uniform distribution within the interval $\left[0,P_{j}^{max}\right]$, as seen in (9). The proposed dual-mode jamming power scheme can increase the uncertainty of Willie’s detection so as to enhance the covert performance.

### 2.3. Channel Model

The link from the airborne node to the ground node can be considered as an LoS link. The flight altitudes of two UAVs are denoted as $H_{a}$ and $H_{j}$ ($H_{a}\neq H_{j}$), respectively. UAV-A transmits covert information to Bob with a prior probability of ρ. The channel gain between UAV-A and Bob or Willie is given by

(10) $$\begin{array}{c} h_{ak}\left[n\right]=\sqrt{\frac{\Omega }{{||a[n]-k||}^{2}+H_{a}^{2}}}, \end{array}$$

where $k\in \left\{b,w\right\}$, Ω is the reference channel gain at 1 m and ||•|| denotes the Euclidean norm.

Similarly, the channel gain between UAV-J and Bob or Willie is given by

(11) $$\begin{array}{c} h_{jk}\left[n\right]=\sqrt{\frac{\Omega }{{||j[n]-k||}^{2}+H_{j}^{2}}}. \end{array}$$

### 2.4. Detection Performance of Willie

The received signal at Willie can be expressed as

(12) $$\begin{array}{c} y_{w}\left[i\right]=\left\{\begin{array}{cc} \sqrt{P_{j0}}h_{jw}x_{j}\left[i\right]+n_{w}\left[i\right], & H_{0}\\ \sqrt{P_{a}}h_{aw}x_{a}\left[i\right]+\sqrt{P_{j1}}h_{jw}x_{j}\left[i\right]+n_{w}\left[i\right], & H_{1}, \end{array}\right. \end{array}$$

where i=1,2,…,m denotes the i-th symbol transmitted during each time slot, and $x_{a}$ and $x_{j}$ correspond to the transmitting signals from UAV-A and UAV-J, respectively. Additionally, $n_{w}\left(i\right)$ denotes the noise, $n_{w}\left[i\right]∽CN\left(0,\sigma_{w}^{2}\right)$.

Willie determines the presence of transmission from UAV-A based on a comparison between its received power $P_{w}$ and a predetermined detection threshold τ. When $P_{w}\geq \tau$, Willie makes a decision $D_{1}$, which indicates that Willie determines that UAV-A is transmitting covert signals. When $P_{w}\leq \tau$, Willie makes a decision $D_{0}$, which indicates that Willie determines that UAV-A is not transmitting covert signals. When m→∞, the average power received by Willie can be given by

(13) $$\begin{array}{c} P_{w}=\left\{\begin{array}{cc} P_{j0}{|h_{jw}\left[n\right]|}^{2}+\sigma_{w}^{2}, & H_{0}\\ P_{a}{|h_{aw}\left[n\right]|}^{2}+P_{j1}{|h_{jw}\left[n\right]|}^{2}+\sigma_{w}^{2}, & H_{1}, \end{array}\right. \end{array}$$

Without loss of generality, assuming equal prior probabilities, Willie’s DEP can be calculated by $\xi \left[n\right]=\frac{1}{2}P_{FA}\left[n\right]+\frac{1}{2}P_{MD}\left[n\right]$. The probability of a false alarm can be given by

(14) $$\begin{array}{cc} P_{FA}\left[n\right] & =\mathrm{Pr}\left(D_{1}|H_{0}\right)=\mathrm{Pr}\left(P_{j0}\left[n\right]{\left|h_{jw}\left[n\right]\right|}^{2}+\sigma_{w}^{2}\geq \tau \right)\\ \; & =\mathrm{Pr}\left(P_{j0}\left[n\right]\geq \frac{\tau -\sigma_{w}^{2}}{|h_{jw}{\left[n\right]|}^{2}}\right)\\ \; & =\left\{\begin{array}{cc} 1, & \tau <\sigma_{w}^{2}\\ 1-\frac{\tau -\sigma_{w}^{2}}{\left(P_{a}\left[n\right]+P_{j}^{\max}\right){\left|h_{jw}\left[n\right]\right|}^{2}}, & \sigma_{w}^{2}\leq \tau \leq \rho_{1}\left[n\right]\\ 0, & \tau >\rho_{1}\left[n\right] \end{array}\right. \end{array}$$

and the probability of missed detection can be given by

(15) $$\begin{array}{cc} P_{MD}\left[n\right] & =\mathrm{Pr}\left(D_{0}|H_{1}\right)=\mathrm{Pr}\left(P_{a}\left[n\right]|h_{aw}{\left[n\right]|}^{2}+P_{j1}\left[n\right]{\left|h_{jw}\left[n\right]\right|}^{2}+\sigma_{w}^{2}\leq \tau \right)\\ \; & =\mathrm{Pr}\left(P_{j1}\left[n\right]\leq \frac{\tau -\sigma_{w}^{2}-P_{a}\left[n\right]{\left|h_{aw}\left[n\right]\right|}^{2}}{|h_{jw}{\left[n\right]|}^{2}}\right)\\ \; & =\left\{\begin{array}{cc} 0, & \tau \leq \rho_{2}\left[n\right]\\ \frac{\tau -\sigma_{w}^{2}-P_{a}\left[n\right]{\left|h_{aw}\left[n\right]\right|}^{2}}{P_{j}^{\max}{\left|h_{jw}\left[n\right]\right|}^{2}}, & \rho_{2}\left[n\right]\leq \tau \leq \rho_{3}\left[n\right]\\ 1, & \tau \geq \rho_{3}\left[n\right], \end{array}\right. \end{array}$$

where $\rho_{1}\left[n\right]=\left(P_{a}\left[n\right]+P_{j}^{max}\right){|h_{jw}\left[n\right]|}^{2}+\sigma_{w}^{2}$, $\rho_{2}\left[n\right]=P_{a}\left[n\right]{|h_{aw}\left[n\right]|}^{2}+\sigma_{w}^{2}$, $\rho_{3}\left[n\right]=P_{j}^{max}{|h_{jw}\left[n\right]|}^{2}+P_{a}\left[n\right]{|h_{aw}\left[n\right]|}^{2}+\sigma_{w}^{2}$.

Obviously, given $P_{a}\left[n\right]$, $P_{j}^{max}$, ${|h_{aw}\left[n\right]|}^{2}$, and ${|h_{jw}\left[n\right]|}^{2}$, ξ[n] can be minimized by optimizing τ at Willie. The optimal detection threshold $\tau^{*}$ and the corresponding minimum ξ[n] can be given by Lemma 1.

**Lemma 1.** 
*With the dual-mode jamming scheme, $P_{j0}\left[n\right]$ and $P_{j1}\left[n\right]$ follow distinct uniform distributions as* (8) *and* (9), given $P_{a}\left[n\right]$, $P_{j}^{max}$, ${|h_{aw}\left[n\right]|}^{2}$, and ${|h_{jw}\left[n\right]|}^{2}$, the optimal detection threshold $\tau^{*}=P_{a}\left[n\right]{|h_{aw}\left[n\right]|}^{2}+\sigma_{w}^{2}$, and the corresponding minimum DEP $\xi^{min}\left[n\right]=\frac{1}{2}\left(1-\frac{P_{a}\left[n\right]{|h_{aw}\left[n\right]|}^{2}}{\left(P_{a}\left[n\right]+P_{j}^{max}\right){|h_{jw}\left[n\right]|}^{2}}\right)$. See Appendix A for detailed proof.

To meet the covert constraint, with an arbitrarily small positive number ε, the minimum detection error probability must satisfy [18]

(16) $$\begin{array}{c} 2\xi^{min}\left[n\right]\geq 1-2\varepsilon ,\;\;\forall n. \end{array}$$

## 3. Covert Rate Maximization

We target to maximize ACR by optimizing the transmit power, flight speeds, accelerations, and trajectories of the dual UAVs. It is formulated as

(17a) $$\begin{array}{cc} P_{1}\;\max_{P_{a}\left[n\right],a\left[n\right],j\left[n\right],V\left[n\right],A\left[n\right]} & \frac{1}{N}\sum_{n=1}^{N}\frac{1}{2}\log_{2}\left(1+\frac{P_{a}\left[n\right]{|h_{ab}\left[n\right]|}^{2}}{P_{j1}{|h_{jb}\left[n\right]|}^{2}+\sigma_{b}^{2}}\right), \end{array}$$

(17b) $$\begin{array}{cc} s.t.\;\;\;\; & (1)∼(7),(16), \end{array}$$

(17c) $$\begin{array}{cc} \; & |V_{a}\left[0\right]|\;=\;|V_{a}\left[N\right]|=V_{\min}, \end{array}$$

(17d) $$\begin{array}{cc} \; & |V_{a}\left[n\right]|\leq V_{\max}, \end{array}$$

(17e) $$\begin{array}{cc} \; & |V_{a}\left[n\right]|\geq V_{\min}, \end{array}$$

(17f) $$\begin{array}{cc} \; & |A_{a}\left[n\right]|\leq A_{\max}, \end{array}$$

(17g) $$\begin{array}{cc} \; & |V_{j}\left[0\right]|\;=\;|V_{j}\left[N\right]|=V_{\min}, \end{array}$$

(17h) $$\begin{array}{cc} \; & |V_{j}\left[n\right]|\leq V_{\max}, \end{array}$$

(17i) $$\begin{array}{cc} \; & |V_{j}\left[n\right]|\geq V_{\min}, \end{array}$$

(17j) $$\begin{array}{cc} \; & |A_{j}\left[n\right]|\leq A_{\max}, \end{array}$$

where $V_{max}$ denotes the maximum flight speed of UAVs, $V_{min}$ denotes the minimum flight speed of UAVs, $A_{max}$ denotes the maximum acceleration of UAVs, $|V_{a}\left[n\right]|$ and $|A_{a}\left[n\right]|$, respectively, denote the speed and acceleration of UAV-A, and $|V_{j}\left[n\right]|$ and $|A_{j}\left[n\right]|$, respectively, denote the speed and acceleration of UAV-J.

The non-convexity of the problem and the significant coupling between the trajectories of the two UAVs render $P_{1}$ challenging to be solved directly. To address this issue, we decompose it into three sub-problems, that is, dynamic trajectory optimization for UAV-A, dynamic trajectory optimization for UAV-J, and optimization for transmit power.

### 3.1. Dynamic Trajectory Optimization for UAV-A

First, by fixing the trajectory of UAV-J and $P_{a}$, we can obtain a sub-problem $P_{2}$

(18a) $$\begin{array}{cc} P_{2}\;\max_{a[n],V[n],A[n]} & \frac{1}{N}\sum_{n=1}^{N}\frac{1}{2}\log_{2}\left(1+\frac{P_{a}\left[n\right]\Omega }{\left(P_{j1}\left[n\right]{|h_{jb}\left[n\right]|}^{2}+\sigma_{b}^{2}\right)\left({||a[n]-b||}^{2}+H_{a}^{2}\right)}\right), \end{array}$$

(18b) $$\begin{array}{cc} s.t.\;\;\; & \frac{P_{a}\left[n\right]\Omega }{2\left(P_{a}\left[n\right]+P_{j}^{max}\right){|h_{jw}\left[n\right]|}^{2}\left({||a[n]-w||}^{2}+H_{a}^{2}\right)}\leq \epsilon , \end{array}$$

(18c) $$\begin{array}{cc} \; & (1),(3),(5),(17c)∽(17f). \end{array}$$

$P_{2}$ continues to present a non-convex optimization challenge. The SCA is employed to address this issue. Initially, introduce a relaxation variable S[n],n=1,2,…,N. Let $S\left[n\right]\geq {||a[n]-b||}^{2}$ and $a^{r}\left[n\right]$ represent the trajectory of UAV-A in the r-th iteration. The corresponding relaxation variable is denoted by $S^{r}\left[n\right]$. Substituting the relaxation variable into the objective function (18a) and conducting the first-order Taylor (FOT) expansion at $S^{r}\left[n\right]$ yields an affine lower bound as

(19) $$\begin{array}{c} \frac{1}{N}\sum_{n=1}^{N}\frac{1}{2}\left[\log_{2}\left(1+\frac{\alpha [n]}{S^{r}\left[n\right]+H_{a}^{2}}\right)-\frac{\alpha \left[n\right](S\left[n\right]-S^{r}\left[n\right])}{ln2\left(S^{r}\left[n\right]+H_{a}^{2}\right)\left(S^{r}\left[n\right]+H_{a}^{2}+\alpha \left[n\right]\right)}\right], \end{array}$$

where $\alpha \left[n\right]=P_{a}\left[n\right]\Omega /P_{j1}\left[n\right]{|h_{jb}\left[n\right]|}^{2}+\sigma_{b}^{2}$.

For the constraint conditions, both (17e) and (18b) exhibit non-convexity. Hence, with FOT approximation, we rewrite (17e) as

(20) $$\begin{array}{c} {|V_{a}^{r}\left[n\right]|}^{2}+2{V_{a}^{r}\left[n\right]}^{T}\left(V_{a}\left[n\right]-V_{a}^{r}\left[n\right]\right)\geq V_{min}^{2}, \end{array}$$

where $V_{a}^{r}\left[n\right]$ is the flight speed of UAV-A obtained in the r-th iteration.

Next, (18b) can be transformed into a convex constraint, which can similarly be written as

(21) $$\begin{array}{c} \frac{1}{2}P_{a}\left[n\right]\Omega -\beta \left[n\right]\left({||a^{r}\left[n\right]-w||}^{2}+2{\left(a^{r}\left[n\right]-w\right)}^{T}\left(a\left[n\right]-a^{r}\left[n\right]\right)+H_{a}^{2}\right)\leq 0, \end{array}$$

where $\beta \left[n\right]=\varepsilon \left(P_{a}\left[n\right]+P_{j}^{max}\right){|h_{jw}\left[n\right]|}^{2}$. Thus, $P_{2}$ can be rewritten as $P_{3}$, which is convex.

(22) $$\begin{array}{cc} P_{3}\;\max_{a[n],S[n],V[n],A[n]}\; & \frac{1}{N}\sum_{n=1}^{N}\frac{1}{2}\left[\log_{2}\left(1+\frac{\alpha [n]}{S^{r}\left[n\right]+H_{a}^{2}}\right)-\right.\\ \; & \left.\frac{\alpha \left[n\right](S\left[n\right]-S^{r}\left[n\right])}{\ln2\left(S^{r}\left[n\right]+H_{a}^{2}\right)\left(S^{r}\left[n\right]+H_{a}^{2}+\alpha \left[n\right]\right)}\right],\\ s.t.\;\;\; & (1),(3),(5),(17c),(17d),(17f),(20),(21),\\ \; & S\left[n\right]\geq {∥a\left[n\right]-b∥}^{2}. \end{array}$$

### 3.2. Dynamic Trajectory Optimization for UAV-J

Secondly, fixing the trajectory of UAV-A and $P_{a}$, a sub-problem can be obtained as

(23a) $$\begin{array}{cc} P_{4}\;\max_{j[n],V[n],A[n]} & \frac{1}{N}\sum_{n=1}^{N}\frac{1}{2}\log_{2}\left(1+\frac{P_{a}\left[n\right]{|h_{ab}\left[n\right]|}^{2}}{P_{j1}\left[n\right]{|h_{jb}\left[n\right]|}^{2}+\sigma_{b}^{2}}\right), \end{array}$$

(23b) $$\begin{array}{cc} s.t.\; & \frac{P_{a}\left[n\right]{|h_{aw}\left[n\right]|}^{2}}{2(P_{a}\left[n\right]+P_{j}^{max})\Omega }\left({||j[n]-w||}^{2}+H_{j}^{2}\right)\leq \varepsilon , \end{array}$$

(23c) $$\begin{array}{cc} \; & (2),(4),(6),(17g),(17h),(17i),(17j). \end{array}$$

Let ${\hat{|h_{ab}\left[n\right]|}}^{2}={|h_{ab}\left[n\right]|}^{2}/\sigma_{b}^{2}$, ${\hat{|h_{jb}\left[n\right]|}}^{2}={|h_{jb}\left[n\right]|}^{2}/\sigma_{b}^{2}$, u[n] be the lower bound of $ln(1+P_{j1}\left[n\right]{\hat{|h_{jb}\left[n\right]|}}^{2}+P_{a}\left[n\right]{\hat{|h_{ab}\left[n\right]|}}^{2})$, and z[n] be the upper bound of $ln(1+P_{j1}\left[n\right]{\hat{|h_{jb}\left[n\right]|}}^{2})$, we can have

(24) $$\begin{array}{c} ln(1+P_{j1}\left[n\right]{\hat{|h_{jb}\left[n\right]|}}^{2}+P_{a}\left[n\right]{\hat{|h_{ab}\left[n\right]|}}^{2})\geq u\left[n\right], \end{array}$$

(25) $$\begin{array}{c} ln(1+P_{j1}\left[n\right]{\hat{|h_{jb}\left[n\right]|}}^{2})\leq z\left[n\right]. \end{array}$$

Therefore, the objective function (23a) is able to be written as $1/N\sum_{n=1}^{N}\left(u\left[n\right]-z\left[n\right]\right)/\left(2ln2\right)$. Given that (24) and (25) are non-convex, we aim to transform the constraints into convex forms. We conduct FTE for $P_{j1}\left[n\right]{\hat{|h_{jb}\left[n\right]|}}^{2}$ at $j^{r}\left[n\right]$; thus, it yields

(26) $$\begin{array}{c} P_{j1}\left[n\right]\gamma \left(\frac{1}{{||j^{r}\left[n\right]-b||}^{2}+H_{j}^{2}}-\frac{{||j[n]-b||}^{2}-{||j^{r}\left[n\right]-b||}^{2}}{{||j^{r}\left[n\right]-b||}^{2}+H_{j}^{2}}\right), \end{array}$$

where $j^{r}\left[n\right]$ is the given feasible point, $\gamma =\Omega /\sigma_{b}^{2}$. Substituting (26) into (24), we can have

(27) $$\begin{array}{cc} \; & 1+P_{a}\left[n\right]\frac{\gamma }{{∥a\left[n\right]-b∥}^{2}+H_{a}^{2}}+P_{j1}\left[n\right]\gamma \left(\frac{1}{∥j^{r}\left[n\right]-b{∥}^{2}+H_{j}^{2}}-\right.\\ \; & \left.\frac{{∥j\left[n\right]-b∥}^{2}-{∥j^{r}\left[n\right]-b∥}^{2}}{(∥j^{r}\left[n\right]-b{∥}^{2}+H_{j}^{2}{)}^{2}}\right)\geq e^{u[n]}. \end{array}$$

Next, we rewrite (25) as

(28) $$\begin{array}{c} 1+P_{j1}\left[n\right]\frac{\gamma }{{||j[n]-b||}^{2}+H_{j}^{2}}\leq e^{z[n]}. \end{array}$$

With the basic inequality ${||j[n]-b||}^{2}+H_{j}^{2}\geq 2H_{j}||j\left[n\right]-b||=q\left[n\right]$, substituting it into (28), we can have

(29) $$\begin{array}{c} 1+P_{j1}\left[n\right]\frac{\gamma }{q[n]}\leq e^{z[n]}. \end{array}$$

We can observe that (29) is non-convex. We conduct the first-order Taylor expansion for $e^{z[n]}$ at $z^{r}\left[n\right]$; thus, it yields

(30) $$\begin{array}{c} 1+P_{j1}\left[n\right]\frac{\gamma }{q[n]}\leq e^{z^{r}\left[n\right]}\left(z\left[n\right]-z^{r}\left[n\right]+1\right), \end{array}$$

where $z^{r}\left[n\right]$ is the given feasible point.

Similar to (20), (17i) can be transformed into ${|V_{j}^{r}\left[n\right]|}^{2}+2{V_{j}^{r}\left[n\right]}^{T}\left(V_{j}\left[n\right]-V_{j}^{r}\left[n\right]\right)\geq V_{min}^{2}$, where $V_{j}^{r}\left[n\right]$ is the flight speed of UAV-J in the r-th iteration. Thus, $P_{4}$ can be rewritten as $P_{5}$, which is a convex problem.

(31) $$\begin{array}{cc} P_{5}\;\max_{j[n],u[n],z[n],q[n],V[n],A[n]}\; & \frac{1}{N}\sum_{n=1}^{N}\frac{\left(u[n]-z[n]\right)}{2\ln2},\\ s.t.\;\;\; & (2),(4),(6),(17g),(17h),(17j),(23b),(27),(30),\\ \; & |V_{j}^{r}{\left[n\right]|}^{2}+2{\left(V_{j}^{r}\left[n\right]\right)}^{T}\left(V_{j}\left[n\right]-V_{j}^{r}\left[n\right]\right)\geq V_{\min}^{2}. \end{array}$$

### 3.3. Optimization for Transmit Power

Fixing the trajectories of UAV-A and UAV-J, we write a sub-problem as

(32) $$\begin{array}{cc} P_{6}\;\max_{P_{a}\left[n\right]}\;\; & \frac{1}{N}\sum_{n=1}^{N}\frac{1}{2}\log_{2}\left(1+\frac{P_{a}\left[n\right]{\left|h_{ab}\left[n\right]\right|}^{2}}{P_{j1}\left[n\right]{\left|h_{jb}\left[n\right]\right|}^{2}+\sigma_{b}^{2}}\right),\\ s.t.\;\; & \frac{P_{a}\left[n\right]{\left|h_{aw}\left[n\right]\right|}^{2}}{2\left(P_{a}\left[n\right]+P_{j}^{\max}\right){\left|h_{jw}\left[n\right]\right|}^{2}}\leq \varepsilon ,\\ \; & (7). \end{array}$$

The objective in (32) is concave. Meanwhile, the conditions imposed on the optimization variable $P_{a}\left[n\right]$ are convex; thus, this problem is convex.

### 3.4. Overall Algorithm

The algorithm steps are given in Table 1. By solving the sub-problems $P_{3}$, $P_{5}$, and $P_{6}$, respectively, and iterating alternately, the transmit power, flight speeds, accelerations, and trajectories of the dual UAVs are jointly optimized.

In terms of the proposed algorithm, sub-problem $P_{3}$ is with the complexity of $O({\left(7N\right)}^{3.5}\log\left(1/\eta \right))$. Sub-problem $P_{5}$ has the complexity of $O({\left(9N\right)}^{3.5}\log\left(1/\eta \right))$. Sub-problem $P_{6}$ has the complexity of $O(N^{3.5}\log\left(1/\eta \right))$. Hence, the total computational complexity of the proposed algorithm is $O(R\left({\left(7N\right)}^{3.5}+{\left(9N\right)}^{3.5}+N^{3.5}\right)\log\left(1/\eta \right))$, where R represents the total number of iterations.

## 4. Numerical Results

A set of rigorous quantitative experiments was conducted using Matlab, with realistic system parameters. The parameters are set as follows. $H_{a}=150$ m, $H_{j}=60$ m, $\delta_{t}=1$ s, $V_{min}=2$ m/s, $V_{max}=25$ m/s, $A_{max}=2$ m/s^2^, Ω=−60 dB, $\sigma_{b}^{2}=\sigma_{w}^{2}=-120$ dB, and $P_{j}^{max}=15$ W.

**Figure 2:**
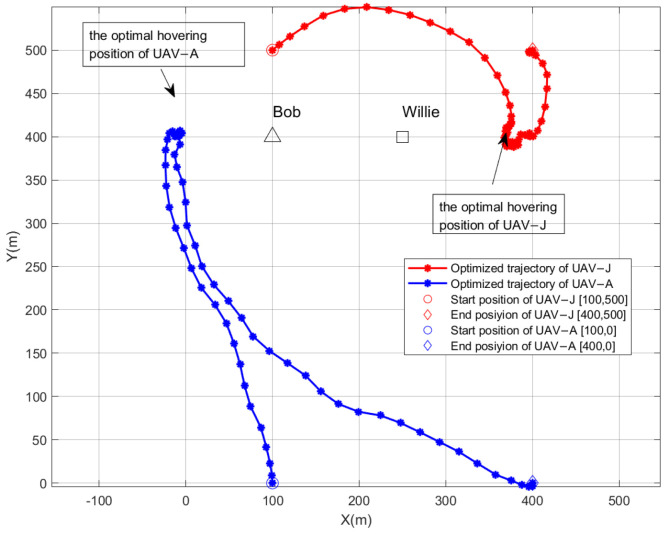
The optimized flight trajectories of the dual UAVs for T = 60 s, when ε=0.05.

Figure 2 demonstrates the optimized flight trajectories of the dual UAVs for T = 60 s. The n-th point in each trajectory denotes the horizontal position of the UAV in the n-th flight time slot. As shown in Figure 2, UAV-A departs from its start position and flies towards Bob. However, the presence of Willie compels UAV-A to gradually veer to the left to evade detection and flies along the left side of Bob. Ultimately, it arrives at the optimal hovering position. After hovering for a duration, UAV-A returns to the end position along the optimized trajectory. Concurrently, UAV-J flies towards Willie from its start position to disrupt the detection of Willie. In order to reduce the jamming to Bob, some distance is maintained between UAV-J and Bob, and then UAV-J arrives at the optimal hovering position. Finally, UAV-J returns to the end position along its optimized trajectory. Such a trajectory route reflects a trade-off between covertness and reliability. The two UAVs deflect and fly as close to the target node as possible, so that the whole system not only meets the requirements of covertness but also ensures the transmission performance of CC.

**Figure 3:**
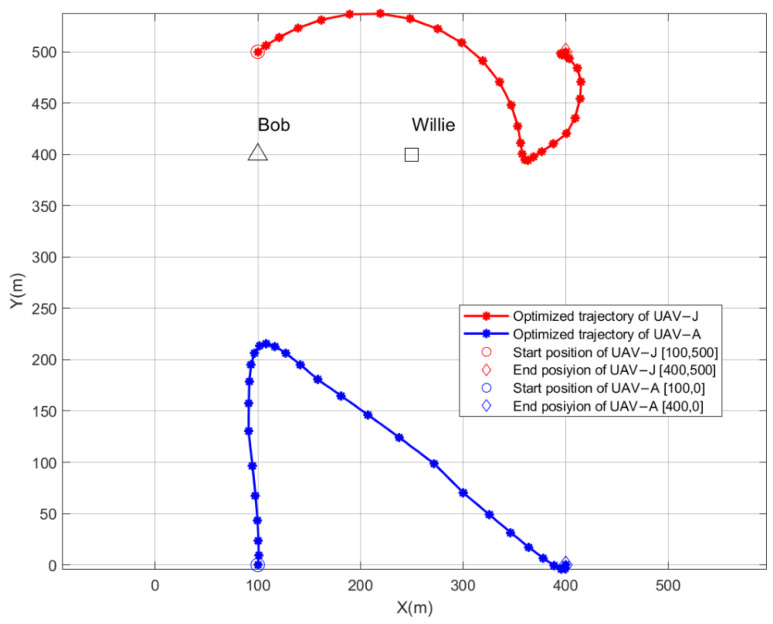
The optimized flight trajectories of the dual UAVs for T = 30 s, when ε=0.05.

Figure 3 demonstrates the optimized flight trajectories of the dual UAVs for T = 30 s. The n-th point in each trajectory denotes the horizontal position of the UAV in the n-th flight time slot. Since the flight duration is limited, UAV-A is unable to arrive at its optimal hovering position. As a fallback, UAV-A attempts to reach a suboptimal hovering position and then returns to the end position by following the optimized trajectory from the hovering position. It implicitly indicates that for a limited flight duration, judiciously increasing the maximum flight speed of UAVs is an effective strategy for enhancing the performance of CC.

**Figure 4:**
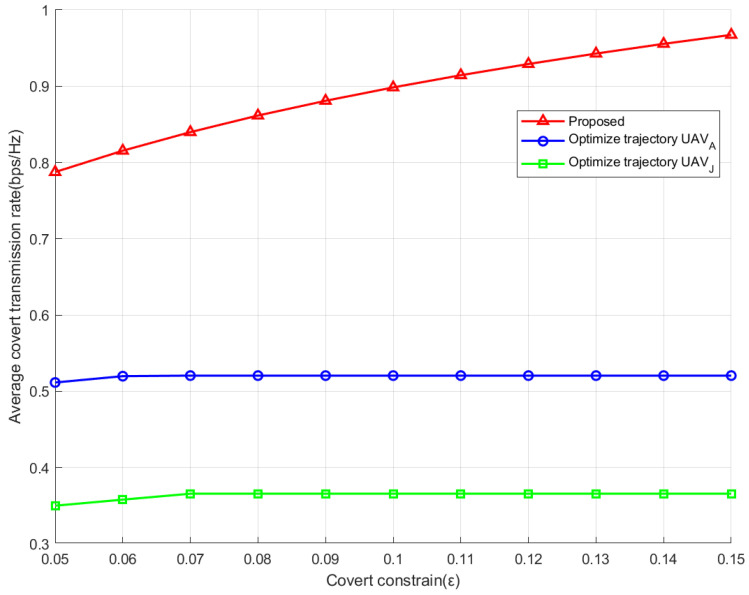
The ACR for various values of ε.

Figure 4 depicts the ACR for various values of ε. As shown, the proposed one outperforms the scheme that optimizes only the trajectory of UAV-A or UAV-J, thereby demonstrating its superiority. The proposed scheme features a jammer and transmitter that can dynamically adjust their positions, and thus, a flexible balance between the covert requirement and transmission rate is achieved. This feature enables the system to achieve a higher covert rate while meeting more stringent covert constraints. ε reflects the strength of the covert constraint. The smaller ε is, the stricter the covert constraint will be. It can be seen that the ACR of the scheme that only optimizes the trajectory of UAV-A or UAV-J exhibits negligible variation as ε increases. However, the ACR of the proposed scheme obviously increases with the value of ε; i.e., as the covert constraint is moderately relaxed, the proposed scheme can obtain more gains in the covert rate.

**Figure 5:**
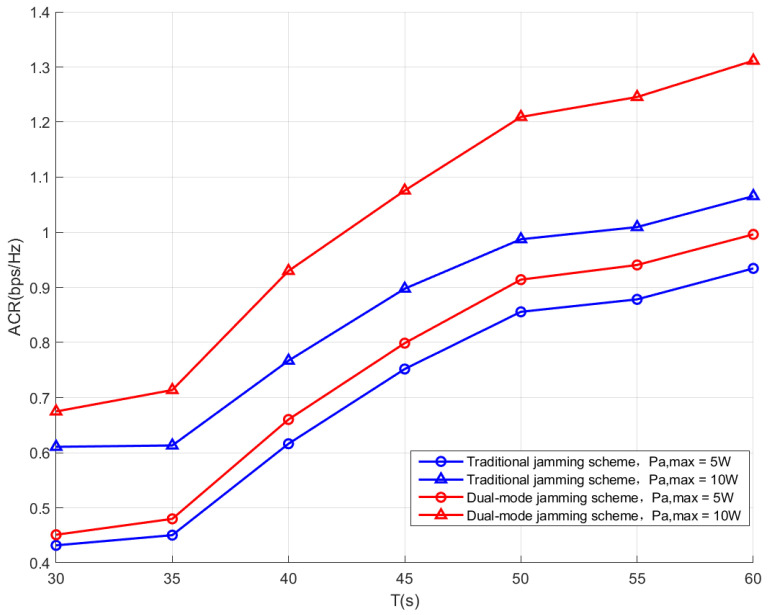
Performance comparison between the proposed dual-mode jamming scheme and the traditional jamming scheme.

Figure 5 depicts the ACRs achieved by the proposed dual-mode jamming scheme and the traditional jamming scheme. Firstly, with the same maximum transmit power, the proposed dual-mode jamming scheme consistently outperforms the traditional jamming scheme through all flight durations. For instance, when T = 60 s, $P_{a}^{max}=10$, the proposed scheme achieves an ACR of approximately 1.3 bps/Hz; however, the ACR achieved by the traditional jamming scheme is only approximately 1.05 bps/Hz. This performance gain arises from the fact that the proposed dual-mode jamming scheme is able to increase the uncertainty in the power received by Willie and thus degrades the accuracy of the detection. Therefore, with the same covert constraint, the proposed scheme can have more opportunities to transmit covert information using higher power. Secondly, with the increase in T, the ACR shows an upward trend, which indicates that as the flight duration becomes more abundant, the UAVs can arrive at the optimal position and hover for a longer duration to achieve better performance. Moreover, with the same maximum transmit power, the performance gap between the two schemes becomes wider with a larger T, which indicates that the proposed dual-mode jamming scheme is particularly suitable for applications in scenarios where UAVs can have long flight durations.

**Figure 6:**
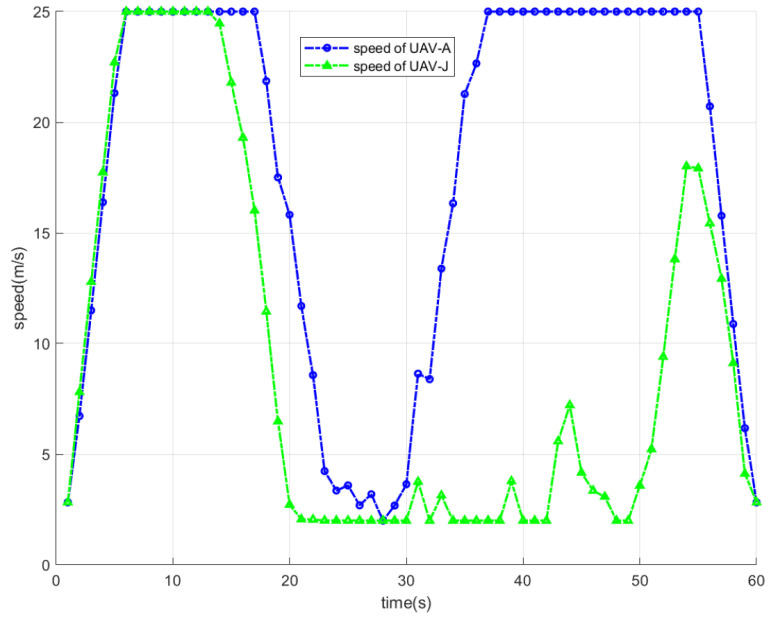
The variation in speed of the dual UAVs within one flight cycle.

Figure 6 shows the variation in speed of the dual UAVs within one flight cycle. It can be observed that both UAVs exhibit a dynamic speed adjustment process tailored to their trajectory optimization. Specifically, UAV-A and UAV-J rapidly accelerate during the start stage towards their respective optimal hovering positions. During this stage, the two UAVs are able to reach their maximum flight speed. As they are getting closer to their optimal hovering positions, they begin to slow down. Notably, when UAV-A and UAV-J have reached their respective optimal hovering positions, their speeds decrease to their lowest point. Obviously, UAV-J will hover for a longer duration than UAV-A. After hovering for a while, UAV-A first begins to accelerate and fly towards the end position. When UAV-A is a certain distance away, UAV-J begins to accelerate towards the end position. Upon reaching the end position, the speeds of both UAVs drop to their lowest point, completing the flight cycle.

## 5. Conclusions

In this paper, CC assisted by dual UAVs is investigated. We propose a dual-mode jamming scheme which can significantly improve the performance. To achieve the maximization of the ACR, we consider an optimization problem which jointly optimizes the transmit power, flight speeds, accelerations, and trajectories of the UAVs. Given the inherent complexity and non-convexity, we develop an efficient iterative algorithm with SCA. Extensive simulations confirm the efficacy of the algorithm in enhancing covert communication performance. In future work, UAV-assisted CC can be combined with integrated sensing and communication, which can efficiently improve the covert performance due to its ability to sense the target.

## Figures and Tables

**Table 1:** Algorithm steps.

Optimal Solution Algorithm
1. Initialization $\left(a^{0},j^{0},P_{a}^{0},S^{0},z^{0},V_{a}^{0},V_{j}^{0}\right)$, index r=0, a given tolerance η;
2. Repeat;
3. Update $\left(a^{r+1},S^{r+1},V_{a}^{r+1}\right)$ by solving problem $P_{3}$ with given $\left(a^{r},j^{r},P_{a}^{r},S^{r},V_{a}^{r}\right)$;
4. Update $\left(j^{r+1},z^{r+1},V_{j}^{r+1}\right)$ by solving problem $P_{5}$ with given $\left(a^{r+1},j^{r},P_{a}^{r},z^{r},V_{j}^{r}\right)$;
5. Update $\left(P_{a}^{r+1}\right)$ by solving problem $P_{6}$ with given $\left(a^{r+1},j^{r+1}\right)$;
6. Update r←r+1;
7. Until the improvement in the objective value is less than η.

## Data Availability

The original contributions presented in this study are included in the article. Further inquiries can be directed to the corresponding author.

## Abbreviations

The following abbreviations are used in this manuscript:

UAV	Unmanned aerial vehicle
SCA	Successive convex approximation
DEP	Detection error probability
ACR	Average covert rate
CC	Covert communication

## Appendix A. Proof of Lemma 1

With the dual-mode jamming scheme, $P_{j0}\left[n\right]$ and $P_{j1}\left[n\right]$ follow distinct uniform distributions as (8) and (9), and then we can calculate $\xi \left[n\right]=\frac{1}{2}P_{FA}\left[n\right]+\frac{1}{2}P_{MD}\left[n\right]$ with (14) and (15). Since the magnitude relationship among the values of $\rho_{1}\left[n\right]$, $\rho_{2}\left[n\right]$, and $\rho_{3}\left[n\right]$ cannot be determined, the discussion can be divided into three cases.

Case 1. When $\rho_{1}\left[n\right]<\rho_{2}\left[n\right]<\rho_{3}\left[n\right]$, with (14) and (15), the DEP ξ[n] can be calculated by

(A1) $$\begin{array}{cc} \xi [n] & =\frac{1}{2}P_{FA}\left[n\right]+\frac{1}{2}P_{MD}\left[n\right]\\ \; & =\left\{\begin{array}{cc} \frac{1}{2}, & \tau <\sigma_{w}^{2}\\ \frac{1}{2}\left(1-\frac{\tau -\sigma_{w}^{2}}{\left(P_{a}\left[n\right]+P_{j}^{max}\right){|h_{jw}\left[n\right]|}^{2}}\right), & \sigma_{w}^{2}\leq \tau <\rho_{1}\left[n\right]\\ 0, & \rho_{1}\left[n\right]\leq \tau <\rho_{2}\left[n\right]\\ \frac{1}{2}\frac{\tau -\sigma_{w}^{2}-P_{a}\left[n\right]{|h_{aw}\left[n\right]|}^{2}}{P_{j}^{max}{|h_{jw}\left[n\right]|}^{2}}, & \rho_{2}\left[n\right]\leq \tau <\rho_{3}\left[n\right]\\ \frac{1}{2}, & \tau \geq \rho_{3}\left[n\right]. \end{array}\right. \end{array}$$

When $\tau <\sigma_{w}^{2}$, ξ[n] is $\frac{1}{2}$. When $\sigma_{w}^{2}\leq \tau <\rho_{1}\left[n\right]$, ξ[n] decreases monotonically as τ. When $\rho_{1}\left[n\right]\leq \tau <\rho_{2}\left[n\right]$, ξ[n]=0, which means the minimum DEP for Case 1 is reached.

Therefore, the optimal threshold for Case 1 is $\tau^{*}\in \left[\rho_{1}\left[n\right],\rho_{2}\left[n\right]\right)$; i.e., the minimum DEP for Case 1 is 0, and thus, it does not meet the covert constraint $2\xi^{\min}\left[n\right]\geq 1-2\varepsilon ,\forall n$. Hence, Case 1 is excluded and is no longer considered in the subsequent optimization process.

Case 2. When $\rho_{2}\left[n\right]<\rho_{1}\left[n\right]<\rho_{3}\left[n\right]$, with (14) and (15), ξ[n] can be calculated by

(A2) $$\begin{array}{cc} \xi [n] & =\frac{1}{2}P_{FA}\left[n\right]+\frac{1}{2}P_{MD}\left[n\right]\\ \; & =\left\{\begin{array}{cc} \frac{1}{2}, & \tau <\sigma_{w}^{2}\\ \frac{1}{2}\left(1-\frac{\tau -\sigma_{w}^{2}}{\left(P_{a}\left[n\right]+P_{j}^{max}\right){|h_{jw}\left[n\right]|}^{2}}\right), & \sigma_{w}^{2}\leq \tau <\rho_{2}\left[n\right]\\ \frac{1}{2}\left(1-\frac{\tau -\sigma_{w}^{2}}{\left(P_{a}\left[n\right]+P_{j}^{max}\right){|h_{jw}\left[n\right]|}^{2}}\right)+\frac{1}{2}\frac{\tau -\sigma_{w}^{2}-P_{a}\left[n\right]{|h_{aw}\left[n\right]|}^{2}}{P_{j}^{max}{|h_{jw}\left[n\right]|}^{2}}, & \rho_{2}\left[n\right]\leq \tau <\rho_{1}\left[n\right]\\ \frac{1}{2}\frac{\tau -\sigma_{w}^{2}-P_{a}\left[n\right]{|h_{aw}\left[n\right]|}^{2}}{P_{j}^{max}{|h_{jw}\left[n\right]|}^{2}}, & \rho_{1}\left[n\right]\leq \tau <\rho_{3}\left[n\right]\\ \frac{1}{2}, & \tau \geq \rho_{3}\left[n\right]. \end{array}\right. \end{array}$$

When $\tau <\sigma_{w}^{2}$, ξ[n] is $\frac{1}{2}$. When $\sigma_{w}^{2}\leq \tau <\rho_{2}\left[n\right]$, ξ[n] decreases monotonically as τ. When $\rho_{2}\left[n\right]\leq \tau <\rho_{1}\left[n\right]$, we can have

(A3) $$\begin{array}{c} \frac{d\xi [n]}{d\tau }=\frac{P_{a}\left[n\right]{|h_{jw}\left[n\right]|}^{2}}{2\left(P_{a}\left[n\right]+P_{j}^{max}\right)P_{j}^{max}{|h_{jw}\left[n\right]|}^{4}}>0. \end{array}$$

Thus, ξ[n] increases monotonically as τ. When $\rho_{1}\left[n\right]\leq \tau <\rho_{3}\left[n\right]$, ξ[n] increases monotonically as τ. When $\tau \geq \rho_{3}\left[n\right]$, ξ[n] is $\frac{1}{2}$.

Therefore, the optimal threshold for Case 2 is $\tau^{*}=\rho_{2}\left[n\right]$, and the corresponding minimum DEP for Case 2 is

(A4) $$\begin{array}{c} \xi^{min}\left[n\right]=\frac{1}{2}\left(1-\frac{P_{a}\left[n\right]{|h_{aw}\left[n\right]|}^{2}}{\left(P_{a}\left[n\right]+P_{j}^{max}\right){|h_{jw}\left[n\right]|}^{2}}\right). \end{array}$$

Case 3. When $\rho_{2}\left[n\right]<\rho_{3}\left[n\right]<\rho_{1}\left[n\right]$, with (14) and (15), ξ[n] can be calculated by

(A5) $$\begin{array}{cc} \xi [n] & =\frac{1}{2}P_{FA}\left[n\right]+\frac{1}{2}P_{MD}\left[n\right]\\ \; & =\left\{\begin{array}{cc} \frac{1}{2}, & \tau <\sigma_{w}^{2}\\ \frac{1}{2}\left(1-\frac{\tau -\sigma_{w}^{2}}{\left(P_{a}\left[n\right]+P_{j}^{max}\right){|h_{jw}\left[n\right]|}^{2}}\right), & \sigma_{w}^{2}\leq \tau <\rho_{2}\left[n\right]\\ \frac{1}{2}\left(1-\frac{\tau -\sigma_{w}^{2}}{\left(P_{a}\left[n\right]+P_{j}^{max}\right){|h_{jw}\left[n\right]|}^{2}}\right)+\frac{1}{2}\frac{\tau -\sigma_{w}^{2}-P_{a}\left[n\right]{|h_{aw}\left[n\right]|}^{2}}{P_{j}^{max}{|h_{jw}\left[n\right]|}^{2}}, & \rho_{2}\left[n\right]\leq \tau <\rho_{3}\left[n\right]\\ \frac{1}{2}\left(1-\frac{\tau -\sigma_{w}^{2}}{\left(P_{a}\left[n\right]+P_{j}^{max}\right){|h_{jw}\left[n\right]|}^{2}}\right)+\frac{1}{2}, & \rho_{3}\left[n\right]\leq \tau <\rho_{1}\left[n\right]\\ \frac{1}{2}, & \tau \geq \rho_{1}\left[n\right]. \end{array}\right. \end{array}$$

When $\tau <\sigma_{w}^{2}$, ξ[n] is $\frac{1}{2}$. When $\sigma_{w}^{2}\leq \tau <\rho_{2}\left[n\right]$, ξ[n] decreases monotonically as τ and when $\rho_{2}\left[n\right]\leq \tau <\rho_{3}\left[n\right]$, ξ[n] increases monotonically as τ, and thus, the minimum DEP for $\sigma_{w}^{2}\leq \tau <\rho_{3}\left[n\right]$ can be obtained when $\tau =\rho_{2}\left[n\right]$, which is $\frac{1}{2}\left(1-\frac{P_{a}\left[n\right]{|h_{aw}\left[n\right]|}^{2}}{\left(P_{a}\left[n\right]+P_{j}^{max}\right){|h_{jw}\left[n\right]|}^{2}}\right)$, less than $\frac{1}{2}$. When $\rho_{3}\left[n\right]\leq \tau <\rho_{1}\left[n\right]$, ξ[n] decreases monotonically as τ and when $\tau \geq \rho_{1}\left[n\right]$, ξ[n] is $\frac{1}{2}$. Hence, based on the above analysis, the optimal threshold for Case 3 is $\tau^{*}=\rho_{2}\left[n\right]$, and the corresponding minimum DEP for Case 3 is $\frac{1}{2}\left(1-\frac{P_{a}\left[n\right]{|h_{aw}\left[n\right]|}^{2}}{\left(P_{a}\left[n\right]+P_{j}^{max}\right){|h_{jw}\left[n\right]|}^{2}}\right)$, which is the same as (A4).

Based on the analysis of these three cases, it can be concluded that the optimal detection threshold $\tau^{*}=P_{a}\left[n\right]{|h_{aw}\left[n\right]|}^{2}+\sigma_{w}^{2}$, and the minimum DEP can be given by (A4).
